# 99529 The Role of ATF6-Mediated Signaling in PARP Inhibitor Resistant Ovarian Cancer

**DOI:** 10.1017/cts.2021.452

**Published:** 2021-03-30

**Authors:** Alexandra, Benjamin Bitler

**Affiliations:** University of Colorado Anschutz Medical Campus

## Abstract

ABSTRACT IMPACT: This work has the potential to identify targetable pathways conveying resistance to PARP inhibitors that may improve ovarian cancer patient outcomes. OBJECTIVES/GOALS: High grade serous ovarian cancer is the deadliest gynecologic malignancy. PARP inhibitors are an FDA approved targeted therapy that is being used more and more frequently in the clinic. It is vital to understand mechanisms driving resistance to this therapy in order to develop treatments to improve patient responses. METHODS/STUDY POPULATION: RNA-sequencing and transcription factor analysis was used to identify pathways of interest. An AP-1 transcriptional reporter assays was used to confirm results of the transcription factor analysis. An unbiased lentiviral shRNA screen was used to identify AP-1 subunits promoting PARP inhibitor resistance. Lentiviral transduction allowed for the knockdown ATF6. Comet assays and two-plasmid systems were used to determine levels of DNA damage and levels of DNA damage repair respectively. RESULTS/ANTICIPATED RESULTS: PARP inhibitor resistant cell lines have increased WNT signaling which promotes to increased DNA damage repair. PARP inhibitor resistant cell lines also have increased AP-1 transcriptional activity, ATF6 expression, and active p38. ATF6 knockdown and p38 inhibition is sufficient to resensitize cells to PARP inhibition. Upon treatment with PARP inhibitors, ATF6 knockdown as well as p38 inhibition lead to increased DNA damage in PARP inhibitor resistant cell lines. RNA-sequencing reveals a signifcant overlap in downregulated genes in cells treated with a β-catenin inhibitor and cells with an ATF6 knockdown. DISCUSSION/SIGNIFICANCE OF FINDINGS: Due to the increasing prevalence of PARP inhibitors in the clinic, it is vital to uncover mechanisms contributing to resistance. This work has the potential to identify targetable pathways conveying resistance to PARP inhibitors that may improve ovarian cancer patient outcomes.

